# Microstructure and Mechanical Properties of Graphene-Reinforced Titanium Matrix/Nano-Hydroxyapatite Nanocomposites

**DOI:** 10.3390/ma11040608

**Published:** 2018-04-16

**Authors:** Feng Li, Xiaosong Jiang, Zhenyi Shao, Degui Zhu, Minhao Zhu

**Affiliations:** 1 School of Materials Science and Engineering, Southwest Jiaotong University, Chengdu 610031, China; lifengswjtu@yeah.net (F.L.); zysao_10227@163.com (Z.S.); dgzhu@home.swjtu.edu.cn (D.Z.); zhuminhao@home.swjtu.edu.cn (M.Z.); 2 Department of Material Engineering, Chengdu Technological University, Chengdu 611730, China

**Keywords:** titanium alloy, nano-hydroxyapatite, graphene, microstructure, mechanical properties

## Abstract

Biomaterial composites made of titanium and hydroxyapatite (HA) powder are among the most important biomedicalmaterials due to their good mechanical properties and biocompatibility. In this work, graphene-reinforced titanium matrix/nano-hydroxyapatite nanocomposites were prepared by vacuum hot-pressing sintering. The microstructure and mechanical properties of graphene-reinforced titanium matrix/nano-hydroxyapatite nanocomposites with different graphene content were systematically investigated. Microstructures of the nanocomposites were examined by X-ray diffraction (XRD), back scattered electron imaging (BSE), scanning electron microscope (SEM) equipped with energy dispersive spectrometer (EDS), electron probe microanalyzer (EPMA), and transmission electron microscope (TEM). The mechanical properties were determined from microhardness, shear strength, and compressive strength. Results showed that during the high-temperature sintering process, complex chemical reactions occurred, resulting in new phases of nucleation such as Ca_3_(PO_4_)_2_, Ti_x_P_y_, and Ti_3_O.The new phases, which easily dropped off under the action of external force, could hinder the densification of sintering and increase the brittleness of the nanocomposites. Results demonstrated that graphene had an impact on the microstructure and mechanical properties of the nanocomposites. Based on the mechanical properties and microstructure of the nanocomposites, the strengthening and fracture mechanisms of the graphene-reinforced titanium matrix/nano-hydroxyapatite nanocomposites with different graphene content were analyzed.

## 1. Introduction

As a commonly used artificial bone graft material, titanium alloys have excellent specific strength, corrosion resistance, durability, and no inflammation or immunogenic response, but it is a bioinert material [1,2]. Simultaneously, it is well known that hydroxyapatite (HA), whose chemical composition is similar to that of human bone, has not only excellent bone osteoconduction ability [3,4,5], but also has the function of increasing the stability of biomaterials [5,6,7,8]. However, poor mechanical properties of pure hyroxyapatite(HA) limits its application [9]. Thus, biocomposites made of titanium alloy and hydroxyapatite have high fracture toughness, excellent bone induction, and osseo integration ability, which are helpful for bone injury to return to normal activities and improve quality of life [10,11]. At present, research on Ti/HA composites at home and abroad have made great progress in material coating. Duarte et al. found that a HA coating prepared by electrochemical deposition was distributed evenly and continuously on the surface of the titanium alloy at low magnification, and showed a porous spherical morphology at high magnification, which facilitates bone integration and drug delivery [7]. However, researchers have recently changed focus to uncoated composites due to the presence of a harmful coating–metal interface layer between Ti/HA coatings. Wang et al. revealed that a Ti-35Nb-2.5Sn/15HA composite had good corrosion resistance and high cytoactivity, which is 40% higher than that of pure titanium [12]. Miranda et al. studied a Ti6Al4V-HA composite prepared by hot pressing and revealed that HA was dispersedly distributed in the matrix [13]. Balbinotti et al. drew a conclusion that the titanium/nano-hydroxyapatite(nHA) composite showed better properties, as the HA could form a more uniform Ca–P deposition, which had better biological activity [14]. Therefore, an uncoated composite can solve the problem of a harmful coating–metal interface layer. Furthermore, titanium can enter the lattice of HA, which further strengthens the bioactivity and biocompatibility of HA. Nevertheless, a Ti/HA composite still has the following problems: (1) mismatch of the elastic modulus leads to the “stress shielding” phenomenon [15,16,17] when the implant is implanted into the human body; (2) severe decomposition and reactions of HA at high-temperature sintering increase the brittleness of composites [14], which would decrease bonding strength that the interface [9] and the strength of composites [18]; and (3) the difference in thermal expansion coefficient between HA and the titanium alloy makes densification difficult to realize [19,20].

In recent years, graphene has been widely used as reinforcement to improve the properties of composites [21,22].For example, Hu et al. investigated the microstructure and properties of a single-layer graphene oxide reinforced titanium-based nanocomposite material and found that oxygen-containing functional groups of graphene oxide partly pyrolyzed during the sintering process, but carbides and oxides were not detected [23].In addition, the nanoindentation hardness (about 11GPa) of the composite was almost three times when compared with that of pure titanium, and the modulus (about 200GPa) was also significantly increased [23]. Song et al. demonstrated the presence of multilayer graphene (MLG) in the composites by Roman spectroscopy and microstructure analysis where it was apparent that an MLG was physically bonded to the matrix, as there was no mesophase formation at the interface [24]. Xu et al. found that although the lubricating property of MLG would disappear when the temperature was higher than 600 °C, the MLG had higheroxygen resistance due to the closed grain boundary, inhibiting the flow of oxygen from the grain boundary [25]. However, in general, the dispersion and integrity of graphene in composites still remain a problem to be solved [26].

Some researchers have shown that lanthanum could form fine oxides in the matrix, which could play the role of dispersion enhancement and improve the plasticity of the composites by reducing the oxidation of the titanium alloy matrix [27,28,29]. Therefore, graphene was used as the nanoscale enhanced phasein this study, and lanthanum was also added to optimize the properties of the titanium alloy/hydroxyapatite nanocomposites. Furthermore, the addition of moderate lanthanum could improve the biological activity of HA [29], and the Ti-29.2Ta-12.2Nb-4.3Zr (wt %) alloy used in the experiment did not contain elements that are harmful to organisms. In this study, graphene-reinforced titanium matrix/nano-hydroxyapatite nanocomposites were prepared by high-energy mechanical ball milling and vacuum hot-pressing sintering. In this case, not only was the problem of the harmful coating–metal interface layer to be solved, but the bioactivity and biocompatibility of HA was enhanced due to the titanium particles entering the lattice of HA. Moreover, by changing the material composition design system, the microstructures of composites were observed and the mechanical properties also determined from microhardness, shear strength, and compressive strength. In addition, the strengthening and fracture mechanisms of the nanocomposites were also analyzed based on the mechanical properties and microstructure of nanocomposites. This is of great significance in promoting the application of titanium/nano-hydroxyapatite composite materials in human hard tissue repair and replacement materials.

## 2. Materials and Methods

**Figure 1:**
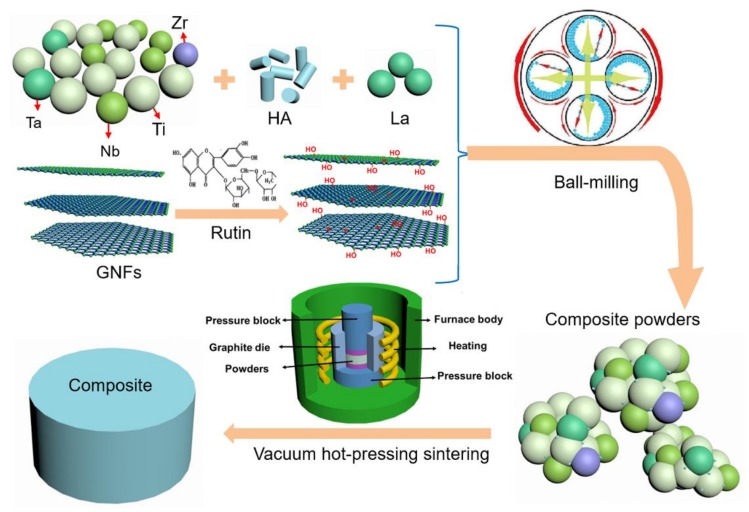
The schematic diagram of the experiment.

The schematic diagram of the experiment is shown in Figure 1. The matrix materials used in this experiment were Ti, Ta, Nb, Zr (300 mesh, purity>99.5%) and HA powders (average diameter size of about 40 nm). Lanthanum and graphene nano-flakes (GNFs) were also added to the nanocomposites, which had acomposition of (54.3Ti-12.2Ta-29.2Nb-4.3Zr)-10HA-0.1La-xGNFs (wt %, x = 0.5, 1.0, 1.5). As shown in Figure 1, the surface modification of graphene was performed by using a rutin solution at the beginning of the experiment [30]. Next, the raw powders were treated by mechanical alloying at a constant speed (380 rpm) for 5 h, and the obtained powders were sintered by a vacuum hot-pressing furnace (VHP-V). The composite was kept for a sintering time of 2 h once it had been heated up to the required temperature, with heating rates of 10 °C/min (up to 800 °C) and 5 °C/min (up to 1000 °C). Furthermore, the pressure was raised to 20 MPa when the temperature was heated up to 1000 °C. After holding the pressure for 1 h, the pressure was released, and the furnace was then naturally cooled to room temperature.

The phase compositions of raw powders, milled powders, and sintered nanocomposites were measured using X-ray diffraction (XRD, X-Pert, PRO-MPD, PANalytical B.V.). The microstructure and composition of the milledpowder and sintered samples were analyzed by scanning electron microscope (SEM, JSM-7001F, JEOL, Japan). Element distribution on the surface of the sintered composite was characterized by an electron probe microanalyzer (EPMA, JXA-8530F, JEOL, Japan) equipped with both an energy dispersive spectrometer (EDS) and wave length dispersive spectrometer (WDS).Transmission electron microscopy (TEM, Tecnai F20ST, FEI, Hillsboro, OR, USA) was used to further analyze the microstructure and phase composition of the sintered nanocomposites. Microhardness of the sintered nanocomposites was tested using a Vickers microhardness tester (Micro-586, Donghua, Shanghai, China). During the testing process, the load was 1 kg, holding time was 15 s, and the testing values of repeated measurements were averaged to improve measurement precision. Compressive and shear strength of the samples were tested with a microcomputer control electron universal testing machine (WDW-3100, Guangzhou guangzhuo precision instrument co. LTD., Guangzhou, China) with a movement rate of 0.5 mm/min. The morphology and characteristics of the fracture surface were analyzed by SEM equipped with EDS.

## 3. Results

### 3.1. Microstructure and Phase Composition of Nanocomposite Powders

#### 3.1.1. XRD Results Analysis

**Figure 2:**
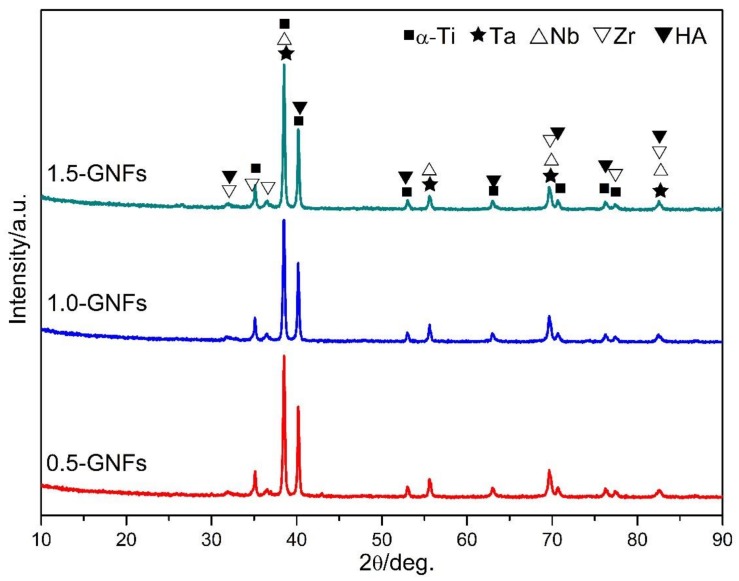
X-ray diffractograms of the milled powders with different contents of graphene nano-flakes (GNFs) (0.5, 1.0, and 1.5 wt %).

The diffraction peaks of Ti, Ta, Nb, Zr, and HA were obviously detected by the XRD spectra of the composite powders (Figure 2). Due to the low content of the added element, the diffraction peaks of La and graphene were not detected. In addition, no additional new phases were found in Figure 2, which indicates that there were no new phases formed in the ball milling process. Bovand et al. found that there were only diffraction peaks of α-Ti in the Ti/nHA composite powders after ball milling, and no peaks were detected or any new phases and HA. This was caused by the fact that HA particles were wrapped by ductile titanium particles [19]. However, Zhao et al. revealed that the phase composition of HA/Ti powders essentially remained unchanged after being milled at different milling times. However, there was a small amount of CaHPO_4_ arising from mechanical alloying of HA during the milling process [20].

#### 3.1.2. SEM Results Analysis

**Figure 3:**
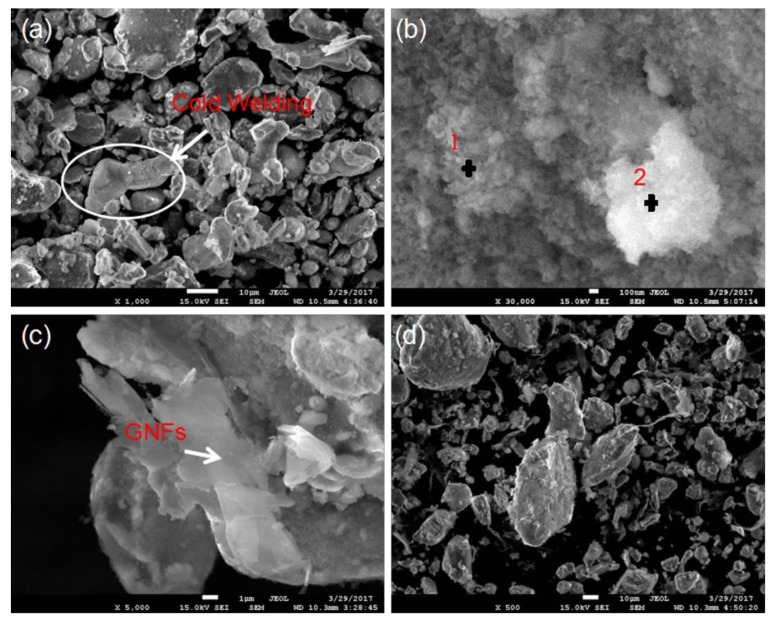
(**a**,**b**) SEM micrographs of the 0.5-GNFsnanocomposites in different regions; and (**c**,**d**) SEM micrographs of the 1.0-GNFs and 1.5-GNFs nanocomposites, respectively.

Figure 3 shows the SEM images of the milled powders, and it can be seen that the milled powder had irregular shapes and different particle sizes ranging from about 3 μm to 40 μm (Figure 3a,d), but the average particle size of the powders in the raw material was 48 μm. This indicated that during the milling process, the collision between powder, grinding balls, and ball mill tank made powder continuously in cold welding and crushing [12], as evidenced by observing the large particle in the white circle (Figure 3a). Theoretically, powders could form large particle aggregates at the beginning of the milling process due to cold welding. Then, with continuous collision, the powders broke and the particle size became smaller owing to the particle hardening effect. Finally, due to the balance between cold welding and fracture action, the particle size reaches a stable value [14,20,31]. There were some white and gray granular aggregates in Figure 3b, and the EDS result (Table 1) showed that these particles were mainly particle agglomerates of HA particles. Compared with most micron-sized particles in the raw materials, nano-HA powders had the tendency to agglomerate together, owing to a larger specific surface area [14,20]. Furthermore, the presence of the metal elements detected in Table 1 might be affected by the metal particles contained in the matrix or agglomerated particles. The topography image of 1.0-GNFs milled powders is shown in Figure 3c. It was apparent that the layered materials (as shown by the white arrow) were GNFs, which were wrapped in other materials and had traces of snapping folds due to powder collision in the mechanical milling process. Similarly, Zhao et al. reported that after being milled for 6 h, the titanium particles became smaller and were present in the form of smaller or larger sheets. Furthermore, a HA/Ti shell structure formed [20], which could be explained as follows. Due to plastic deformation, the titanium particles became flaky at the beginning of the ball mill process. With the increase of milling time, it was broken into smaller flakes under the effect of work hardening. Then, small HA particles with high surface energy were adhered to the surface of the titanium sheet [20]. 

### 3.2. Microstructure and Phase Analysis of the Sintered Nanocomposites

#### 3.2.1. XRD Results Analysis

**Figure 4:**
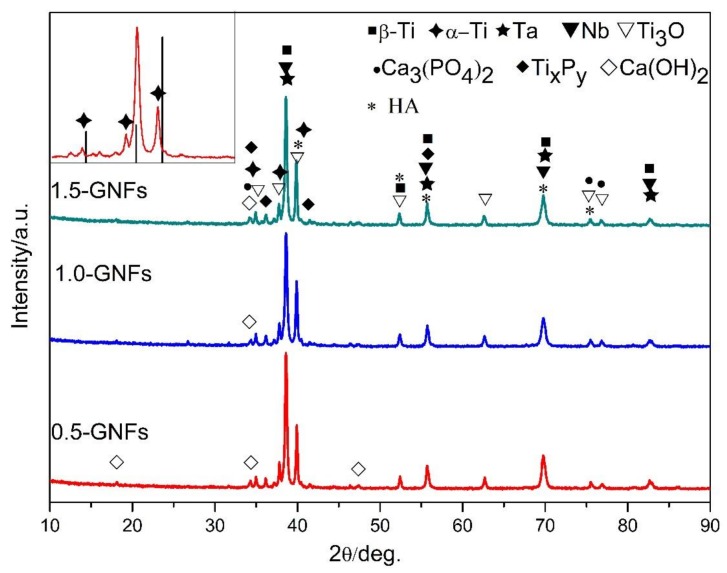
X-ray diffraction patterns of nanocomposites sintered at 1000 °C. A comparison with α-Ti standard card (44-1294) is shown in the upper left panel.

The XRD pattern of the sintered composite is shown in Figure 4. It is apparent that complex reactions occurred during high-temperature sintering, as Ca_3_(PO_4_)_2_, Ti_x_P_y_, Ti_3_O, α-Ti, β-Ti, Ta, Nb, Ca(OH)_2_, and HA were detected. The titanium element in the sintered composites was in the form of α-Ti, β-Ti, and Ti_3_O, and α-Ti was the form of titanium in the raw materials. The β-Ti was formed by the phase transition of titanium during the sintering process, and the existence of the β-phase with lower elastic modulus was beneficial in decreasing the elastic modulus of the composites. The presence of Nb, a β-type stabilizing element, made it possible to retain a large amount of β-Ti after sintering. Titanium oxides might be formed by the reaction between titanium and residual oxygen, but Arifin et al. and He et al. reported that titanium oxide (TiO_2_) was formed by the reaction between the titanium and water vapor produced from the decomposition of HA [32,33]. Furthermore, the reaction products did not contain other new phases including tantalum or niobium (except for the metal element of Ta and Nb), which indicated that the powder particles of Ta and Nb did not react during sintering.

#### 3.2.2. SEM Results Analysis

**Figure 5:**
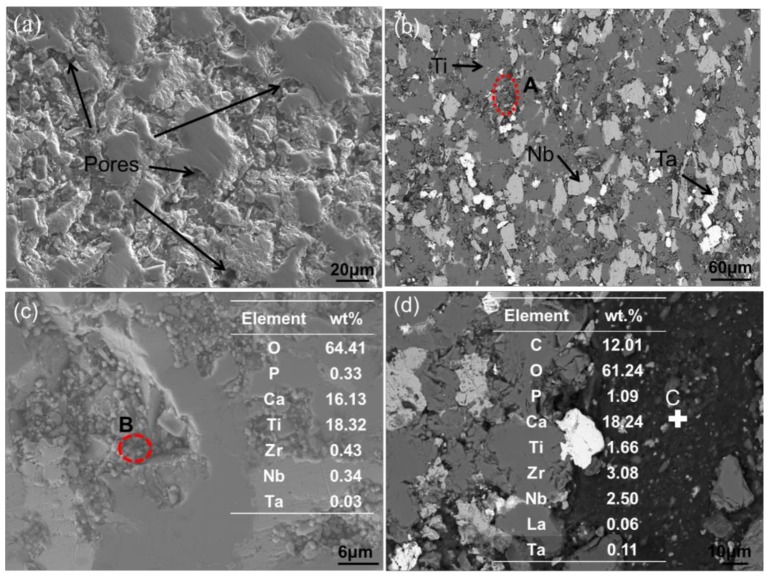
(**a**,**b**) SEM micrographs of the 0.5-GNFs nanocomposite in different regions; and (**c**,**d**) SEM micrographs of the 1.0-GNFs and 1.5-GNFs nanocomposites, respectively.

**Figure 6:**
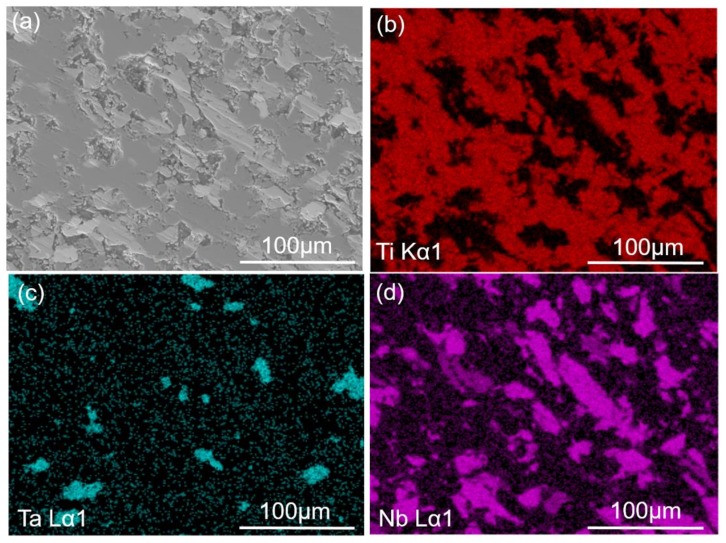

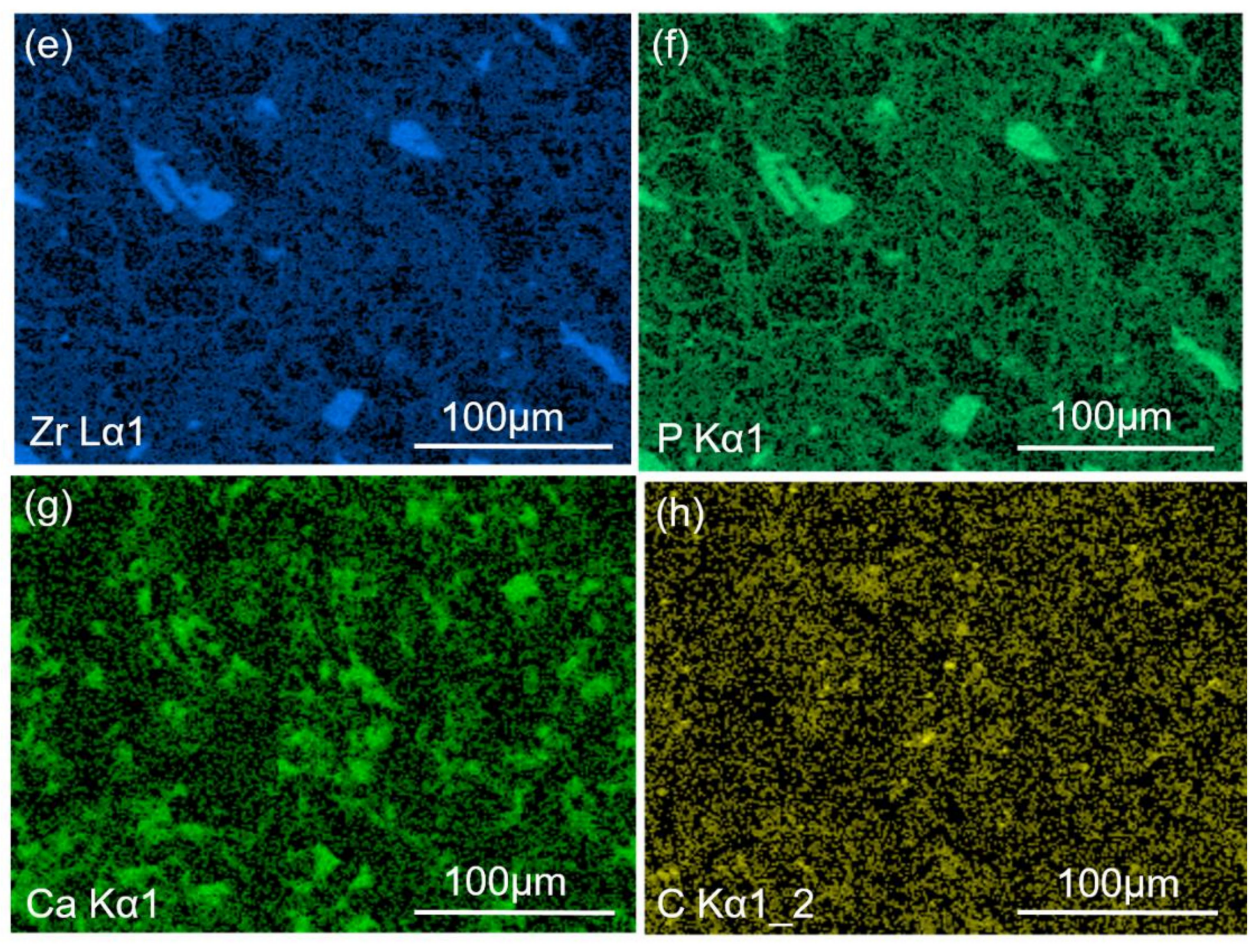
(**a**) SEM micrographs of the 0.5-GNFs nanocomposite sintered at 1000 °C. The corresponding mapping scanning results of elements: (**b**) Ti; (**c**) Ta; (**d**) Nb; (**e**) Zr; (**f**) P; (**g**) Ca; and (**h**) C.

The SEM and EDS results of the sintered composites are shown in Figure 5, where Figure 5a is the secondary electron topography of the 0.5-GNFs composite. It can be seen that the surface of the sample was not flat and there were a large number of pores, indicated by the white arrows (Figure 5a) at the interface, which could indirectly reflect the lower density of the sintered composites. Figure 6b shows the back scattered electron image of the sintered 0.5-GNFs composite, and it was apparent that the composition distribution of the 0.5-GNFs composite after sintering was relatively uniform. Furthermore, the surface of the sintered 0.5-GNFs composite was mainly composed of three regions containing different colors. Gray-black areas (77.96Ti-13.49O-8.26C wt %) were the largest and main enrichment areas of titanium. Fragmentation areas of gray-black (spot A, 54.51Ti, 20.37O, 13.61C, 4.85Ca, 3.74P, 1.21Nb, 1Zr wt %) were also enrichment areas of titanium, but the titanium content of the fragmentation areas was lower than that of the gray-black regions. White regions (74.15Ta, 17.5C, 6.37O wt %) were enrichment areas of Ta, and gray areas (71.9Nb, 17.65C, 5.13Zr, 4.96O wt %) were enrichment areas of Nb. Wang et al. found gray enrichment regions of titanium and white enrichment regions of Nb in the Ti-35Nb-xSn/15HA biocomposite [16].Figure 5c showed that there were some particles that were mainly decomposition products of HA in the fragmentation areas. It could also be seen that there was an area including the shedding phase which was mainly the accumulation of the Ca–P phases in Figure 5d. Surface scan images of the 0.5-GNFs composite are shown in Figure 6. It can be seen that the Ti and Nb elements in the composites were basically complementary to each other and comprised the body of the composite. Ta and Zr elements were distributed in their gaps, and C and Ca elements were distributed across the entire plane. Some Ca elements were generally enriched around the matrix elements and the Ca element was also distributed in the enrichment areas of Ti, Nb, Ta, and other elements.

#### 3.2.3. EPMA Results Analysis

**Figure 7:**
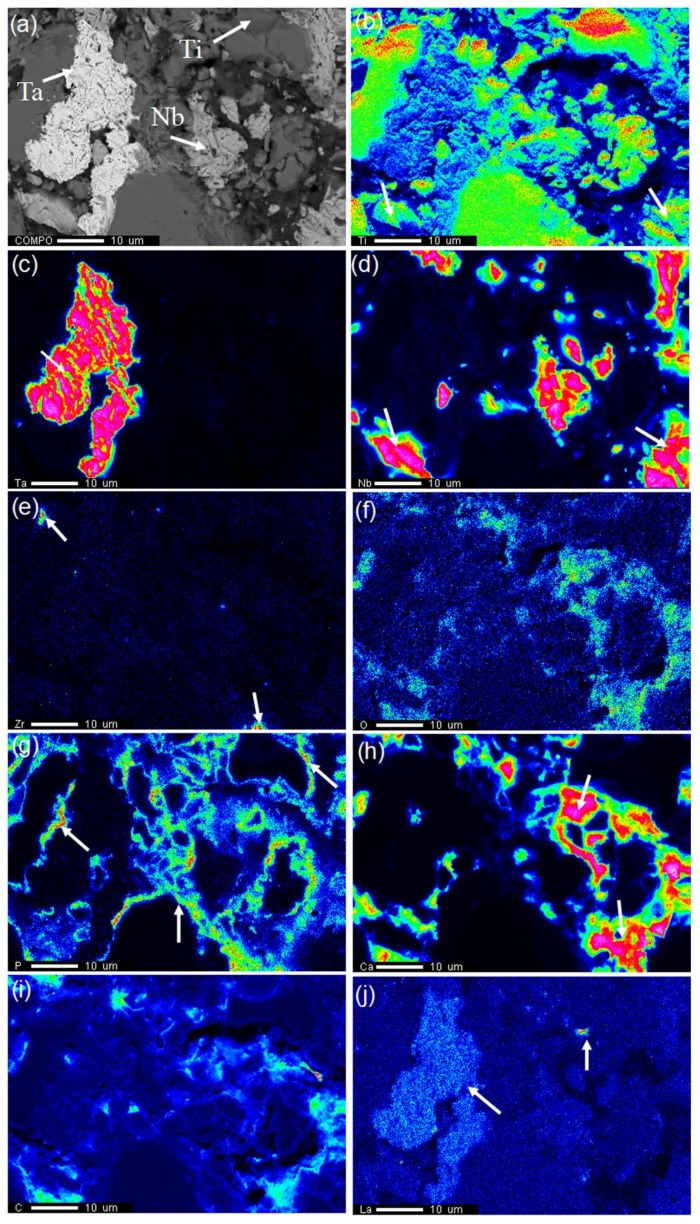
(**a**) SEM micrographs of the 0.5-GNFs nanocomposite sintered at 1000 °C. The corresponding mapping scanning results of electron probe microanalyzer (EPMA): (**b**) Ti; (**c**) Ta; (**d**) Nb; (**e**) Zr; (**f**) O; (**g**) P; (**h**) Ca; (**i**) C; and (**j**) La.

Elements distribution characterized by EPMA on the surface of the 0.5-GNFs composite is shown in Figure 7, and Figure 7a is the backscattered electron image of the 0.5-GNFs composites. It can be seen that titanium was distributed almost across the entire surface in Figure 7b, and the completely gray areas in Figure 7a are the enrichment areas of titanium. Some regions (indicated by arrows) of titanium element in Figure 7b coincided with the partially enriched regions of Nb (Figure 7d). As shown in the above analysis, white regions were the enrichment regions of Ta, and the gray regions were the enrichment regions of Nb. The distribution of the Ta and Nb elements in Figure 7c,d also proved this conclusion. There were only two enrichment regions of Zr in Figure 7e, which might be due to the relatively low content of Zr and its almost uniform distribution. Combining the distribution of O, P, and Ca elements in Figure 7f–h, it could be seen that their enrichment areas had large overlaps (indicated by the arrows in Figure 7h) and were distributed in the gaps of Ti, Ta, and Nb. This indicated that the decomposition or reaction products of HA, such as Ca_3_(PO_4_)_2_, were distributed in the matrix. Balbinotti et al. found that phases containing Ca and P in the Ti/HA composite were distributed around the titanium matrix [14]. In addition, it could be seen that some enrichment regions of P were adjacent to the partial enrichment regions of Ti from the areas indicated by the arrows in Figure 7g. This might be the distribution of the Ti_x_P_y_ compound in the XRD results. Distributions of the C and La elements were shown in Figure 7i,j, respectively, and their distribution areas were mainly blue and light blue. The reunion areas of C and phosphorus–calcium phases were similar and distributed in the gaps of the matrix. The largest enrichment region of La coincided with the enrichment region of tantalum.

#### 3.2.4. TEM Results Analysis

**Figure 8:**
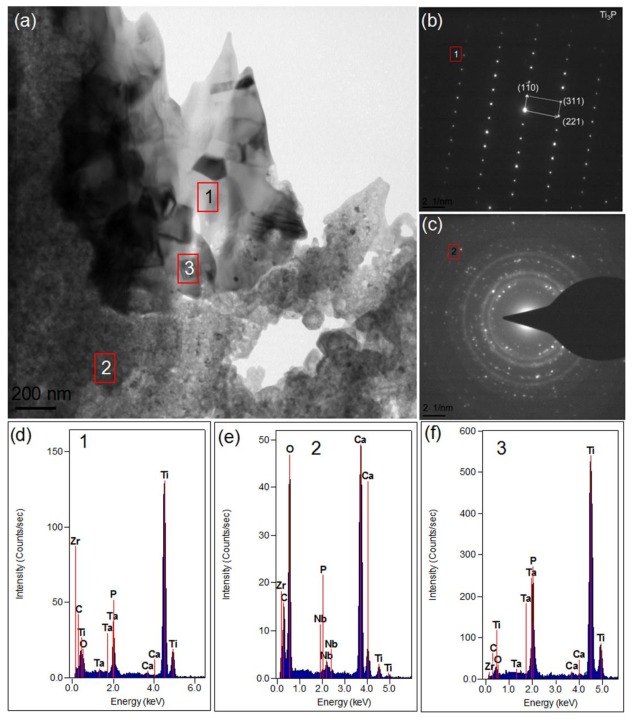
(**a**) TEM image of the 1.5-GNFs nanocomposite sintered at 1000 °C; (**b**,**c**) the corresponding selected area diffraction (SAD) patterns of spots 1and 2; and (**d**–**f**) the corresponding EDS spectrums of spots 1–3.

**Figure 9:**
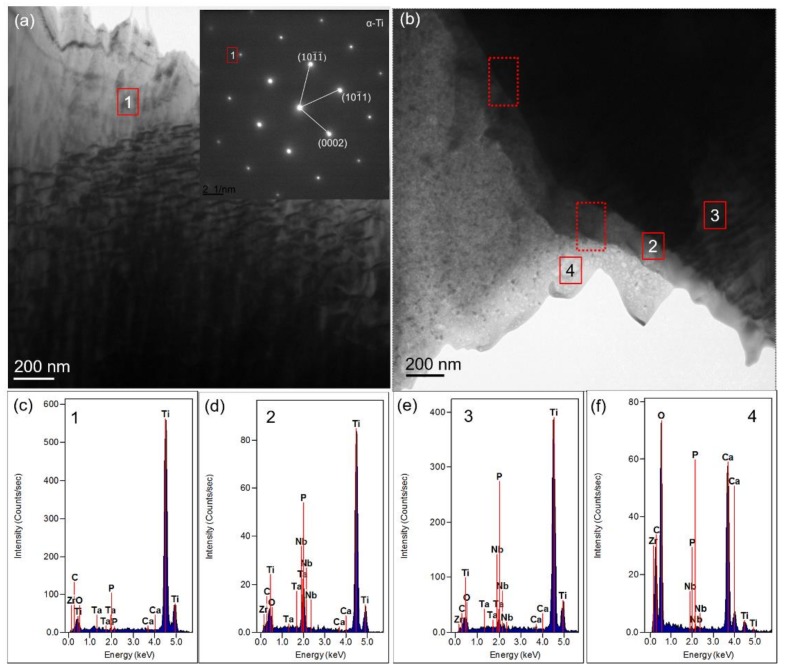
(**a**,**b**) TEM image of the 1.5-GNFs nanocomposite sintered at 1000 °C in different areas. The inset shows the SAD pattern of spot 1; and (**c**–**f**) the corresponding EDS spectrums of spots 1–4.

Figure 8 shows the TEM images of the 1.5-GNFs composites prepared by vacuum hot pressing. It could be seen that, considering the mass thickness contrast, the shading differences between spots 1 and 3 in Figure 8a were caused by the thickness of the sample. It is known that the phase of spot 1 was Ti_3_P, according to the selected area diffraction pattern of spot 1 in Figure 8b. The diffraction pattern of spot 2 in Figure 8c showed that it contained composite crystals and amorphous phases [34], and the discontinuity of the diffractive ring was caused by a large grain or small grain number. It was apparent that phases of spot 2 were mainly composed of Ca, O, C, and Zr by corresponding EDS spectrums. In addition, there were small amounts of Ti and Nb detected. Furthermore, it was seen that the phases of point 2 mainly contained CaO, CaCO_3_, CaZrO_3_, and so on by analyzing the selected area diffraction spectrum(Figure 8c) [20].The detected elements of spot 3 mainly contained Ti, P, O, Ca, and so on, which was similar to the element distribution of spot 1. The TEM image in Figure 9a and the corresponding EDS results of spot 1 in Figure 9c indicated the presence of residual α-Ti in the sintered composite. Phases of points 2 and 3 (Figure 9b) were mainly composed of Ti and also contained O, Ca, and P. Phases of point 4 (Figure 9f) were mainly composed of O, Ca, C, and Zr, and the element distribution of spot 4 (Figure 9f) was similar to that of point 2 (Figure 8). It was seen that there were no obvious cracks or pores at the interface of the composite by observing areas in the dashed box (Figure 9c). Chang et al. studied the TEM images of the composite and observed that although there were some pores, the interface still had a good combination [9].

### 3.3. Mechanical Properties of the Sintered Nanocomposite

**Figure 10:**
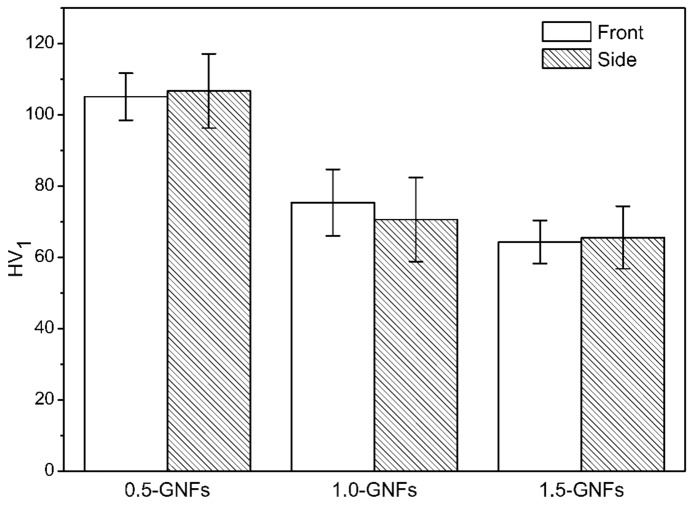
Themicrohardness of the nanocomposites sintered at 1000 °C with different contents of GNFs.

**Figure 11:**
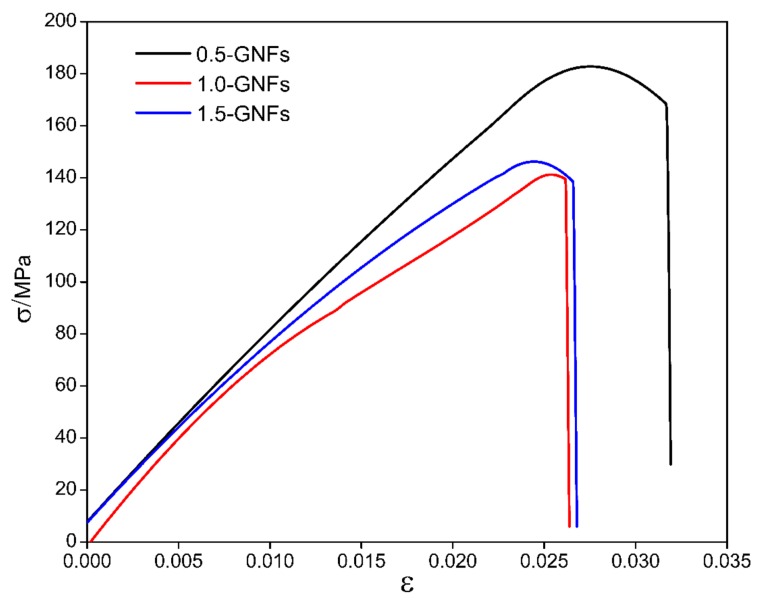
Compressive stress–strain curves of nanocomposites sintered at 1000 °C with different contents of GNFs.

Figure 10 shows the microhardness of the sintered composites, and the results showed that the frontal and lateral hardness of the samples decreased with the increase of graphene content. Table 2 and Figure 11 show the results and comparison of the mechanical properties in the sintered composites. It was seen that the compressive strength and shear strength of the 0.5-GNFs composite were the highest by comparing the results in Table 2. Similarly, Song et al. found that the hardness and elastic modulus of 0.5 wt % MLG/Ti composite were increased when compared with pure titanium. However, the hardness and elastic modulus of the 1.5 wt % MLG/Ti composite decreased, and yield strength also had a similar trend [24]. Hu et al. reported that the content of graphene oxide added to the composite had an optimum value. Although graphene oxide (SLGO) had the role of bearing loads and fixing dislocation movement, when deformation began, its hardness would decrease due to the reunion of SLGO when the amount of SLGO reached a certain degree [23].

### 3.4. Fracture Surface Analysis of the Sintered Nanocomposites

**Figure 12:**
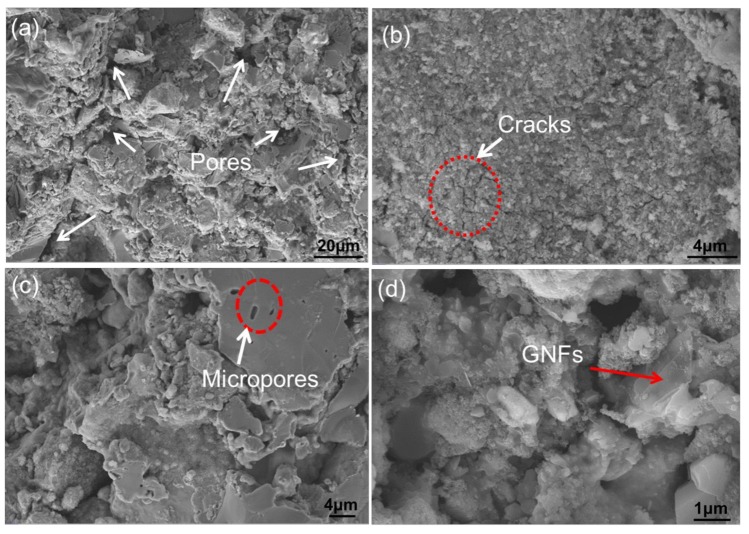
SEM micrographs of shear fracture surfaces of (**a**,**b**) the 0.5-GNFs nanocomposites in different regions; (**c**) the 1.0-GNFs nanocomposites; and (**d**) the 1.5-GNFs nanocomposites, respectively.

Figure 12 shows the SEM micrographs of the shear fracture surfaces of the sintered composite, and the shear fractures of the 0.5-GNFs composite are shown in Figure 12a,b. Many grainy agglomerates and plate-like substances on the uneven fracture surface were seen, and there were many pores of different sizes between the plate-like substances. This showed that the density of the sintered sample was not very high, which was mainly caused by the escaped gas generated during sintering and the difference in the thermal expansion coefficient between the titanium alloy and HA. Cracks might form during cooling due to the difference inthermal expansion coefficient between the matrix and hard phases produced by the reactions of HA. Then, the cracks would spread under the action of external force, which made organization around the cracks easily drop off from the matrix. This was confirmed by the presence of a large number of tiny cracks in the white circle, which can be seen in Figure 12b. Shear fractures images of the 1.0-GNFs composite are shown in Figure 12c. Micropores on the surface of the lamellar material were caused by neck shrinkage during the sintering process because the number of micropores was reduced in the process of necking, and the shape of the micropores was regularized. SEM images of the shear fracture surfaces of the 1.5-GNFs composite are shown in Figure 12d, and the transparent layered material in Figure 12d was graphene. In addition, the graphene would overlap due to the characteristics of a high specific surface area, and might be broken by shearing forces. Song et al. reported that when the graphene content was very high, multilayer graphene agglomeration led to weak bonds between the interface and poor stress transfer, which resulted in the decrease of mechanical properties [24].

**Figure 13:**
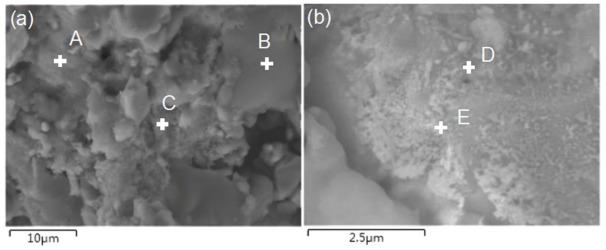
SEM micrographs of shear fracture surfaces of (**a**) 1.0-GNFs; and (**b**) 1.5-GNFs nanocomposites sintered at 1000 °C, respectively.

Figure 13 shows the SEM images of the shear fracture surface, and the corresponding EDS result is shown in Table 3. It could be seen that the shear fracture surface of the 1.0-GNFs composite was mainly composed of an amorphous phase, lamellar material, and granular agglomeration by observing Figure 13a. It was believed that the mixture (region A) of the granular agglomeration and amorphous phase was mainly composed of the decomposition products of HA and Nb, according to the results in Table 3. The lamellar material (region B) was mainly titanium and titanium oxides, and there were some decomposition and reaction products of HA on the surface. Granular agglomeration (zone C) was mainly composed of reaction products of HA and lanthanum, and a small amount of metal particles were mixed in the agglomeration. Similar morphologies were also found by other researchers, and A et al. revealed that the melting points of HA and the titanium alloy was different, which resulted in the mixing of HA and molten titanium under high temperature [33]. Phases of spots D and E in Figure 13b were mainly composed of Ta and some decomposition products of HA. Therefore, phases containing Ca and P might be mixed with other powders to form a granular agglomeration or amorphous phase, and might also be distributed on the surface of the alloy matrix during sintering. The decomposition or reaction products distributed in the gap were brittle and tended to cause intergranular fracture after sintering, which was similar to that found by Balbinotti et al. [14].

## 4. Discussion

Research shows that HA would first dehydrate to produce anoxic hydroxyapatite (OHA) during the high-temperature sintering process [35]. The possible decomposition reactions are shown in Equations (1) and (2) [8,33], and the difference lay in the formation of calcium oxide. Ca(OH)_2_ detected by XRD (Figure 4) was generated by the reaction between calcium oxide and water. The sources of water used as a reactant were as follows: (1) reaction products during sintering [36]; and (2) water in the preparation process of the sample. Furthermore, thermodynamic analysis (∆G > 0) at 1000 °C revealed that water generated by the decomposition reaction was in the form of vapor and did not decompose to produce hydrogen and oxygen.TiO_3_ detected by XRD (Figure 4) was generated by the reaction between Ti and oxygen in the environment. Additionally, the diffraction peaks of residual α-Ti detected by XRD (Figure 4) moved left when compared with α-Ti standard card (44-1294), which is shown in the upper left panel of Figure 4. As other elements of larger radius entered the crystal lattice of titanium, this could make the lattice constant become larger and the angle become smaller. Furthermore, the grain size of phases was calculated by XRD Pattern Processing and Identification software. The result is shown in Table 4, and it is apparent that the angles of the three peaks for α-Ti after sintering were lower than before sintering. The calculation results (Table 4) showed that the grain size of α-Ti significantly increased after sintering, which effectively proved this point.

(1) $$Ca_{10}{\left(PO_{4}\right)}_{6}{\left(OH\right)}_{2}=Ca_{10}{\left(PO_{4}\right)}_{6}{\left(OH\right)}_{2-2x}O_{x}+xH_{2}O(g)$$

(2) $$Ca_{10}{\left(PO_{4}\right)}_{6}{\left(OH\right)}_{2}=Ca_{10-x}{\left(PO_{4}\right)}_{6}{\left(OH\right)}_{2-2x}+xCaO+xH_{2}O(g)$$

(3) $$2H_{2}O(g)=2H_{2}(g)+O_{2}(g)\;\Delta G=84.849k\mathrm{cal}$$

In addition, the sintered nanocomposite had decomposed to a certain extent at 1000 °C when compared with the relatively stable pure HA due to the existence of Ti and other metal elements [37]. Ye et al. also found that due to the presence of Ti, dehydroxylation and decomposition of HA began at 800 °C [8]. Ca_3_(PO_4_)_2_ detected by XRD (Figure 4) was one of the decomposition products of HA or hypoxic hydroxyapatite [27,28]. Some of the literature has suggested that the decomposition reactions of producing Ca_3_(PO_4_)_2_ are shown in Equations (4) and (5) [27,28]. However, Nath et al. revealed that Ca_3_(PO_4_)_2_ may be formed by the reaction between HA and titanium oxide, as shown in Equations (6) and (7). Furthermore, thermodynamic analysis (∆G < 0) revealed that the reaction (Equation(7)) could be carried out when the temperature was higher than 720 °C [38]. Based on the result of the XRD (Figure 4) and TEM (Figure 8), Equations (8) and (9) are the chemical reactions in this study which have been inferred from literatures [14,15,16,17,18,19,20,21,22,23,24,25,26,27,28,29,30,31,32,33,34,35,36,37,38]. Additionally, Ti_x_P_y_ detected by XRD (Figure 4) was formed by the reaction between titanium and phosphorus which was derived from Equations (10) to (11). He et al. reported that Ti_x_P_y_ was formed by the reaction between titanium and phosphorus which was derived from Equation (10) [32]. In addition, thermodynamic analysis (∆G < 0) revealed that the reaction (Equation (10)) could be carried out at 1000 °C. However, some researchers believed that phosphorus isderived from Equation (11) [14]. It was found that the angles of the diffraction peaks for Ta and Nb were almost unchanged before and after sintering, but grain size was obviously reduced by comparing the results (Table 4). This was caused by the compacting process of the particles during vacuum hot-pressing sintering, such as particles gliding across each other, grain crushing, grain rearranging, and so on. Furthermore, the grain growth rate was reduced after recrystallization due to the existence of La and other particles, and this would have further led to the decrease of grain size after recrystallization.

(4) $$Ca_{10}{\left(PO_{4}\right)}_{6}{\left(OH\right)}_{2-2x}O_{x}=2Ca_{3}{\left(PO_{4}\right)}_{2}+Ca_{4}P_{2}O_{9}+(1-x)H_{2}O(g)$$

(5) $$Ca_{10}{\left(PO_{4}\right)}_{6}{\left(OH\right)}_{2}=2Ca_{3}{\left(PO_{4}\right)}_{2}+Ca_{4}P_{2}O_{9}+H_{2}O(g)$$

(6) $$Ti+O_{2}(g)=\mathrm{Ti}O_{2}$$

(7) $$Ca_{10}{\left(PO_{4}\right)}_{6}{\left(OH\right)}_{2}+\mathrm{Ti}O_{2}=3Ca_{3}{\left(PO_{4}\right)}_{2}+\mathrm{CaTi}O_{3}+H_{2}O(g)$$

(8) $$Ca_{10}{\left(PO_{4}\right)}_{6}{\left(\mathrm{OH}\right)}_{2}+\mathrm{Ti}{+3/2O}_{2}(g)=3Ca_{3}{\left(PO_{4}\right)}_{2}+\mathrm{CaO}+\mathrm{Ti}O_{3}+H_{2}O(g)$$

(9) $$\mathrm{CaO}+H_{2}O(g)=\mathrm{Ca}{\left(\mathrm{OH}\right)}_{2}$$

(10) $$6\mathrm{Zr}+O_{2}(g)+2Ca_{3}{\left(PO_{4}\right)}_{2}=6\mathrm{CaZr}O_{3}+4P∆G=-518.349\;\mathrm{kcal}$$

(11) $$\mathrm{Ti}+1/2O_{2}(g)+Ca_{3}{\left(PO_{4}\right)}_{2}=3\mathrm{CaTi}O_{3}+2P$$

It can be seen that there were some pores, indicated by the white arrows on the surface of the sample (Figure 5a), which could indirectly reflect the density of the sintered composites. In fact, the density of the composites obtained by drainage method is, respectively, 78.47(0.5-GNFs), 77.21(1.0-GNFs), and 77.41(1.5-GNFs). With the change of graphene content, the density difference of composites is very small. In addition, it can be seen from Figure 5a that the sizes of the majority of pores are about 3.4–10 μm. And the pores are mainly distributed between the matrix and the reaction phases, which is because the pores are mainly caused by the escape of the gas produced by the reactions and the difference in thermal expansion coefficient between different phases. Similar pores (0.2–16 μm) can be easily observed from Figure 13a in that their unique pore structure can give them the characteristic of ultralow density and large surface area for a human body with structural and functional integration. 

As the enrichment regions of Ti, Ta, Nb, and so on in Figure 5b contained other elements, there was the presence of illustrated inter-diffusion between the elements during the sintering process, which has also been drawn from results of the EDS in Figure 8d–f. Researchers have also reported that oxygen and titanium elements in the HA/Ti composites were mutually diffused [29]. Oxygen entered the titanium lattice as gap atoms at the beginning of diffusion, and titanium oxide formed when oxygen was saturated in the titanium lattice, which resulted in the decrease of diffusion rate [32]. It was seen that the reduction of P in the shedding phase (Figure 5d) was due to the loss of phosphorus-containing phases. Therefore, this indicated that during the sintering process, interfacial bonding between the hard-brittle phases formed by the reactions and matrix were weak, and the hard-brittle phases easily dropped off under the action of external force. Balbinotti et al. reported that the calcium-containing phases formed between the titanium particles were brittle phases in the Ti/HA composites. Furthermore, the phases easily fell off from the material during mechanical polishing, which would lead to the formation of the undulating plane, cracks, and pores between titanium particles [14]. It was seen that the difference between the distribution of P and Ca might be due to the disengagement of partial phases by observing the surface scan images (Figure 6). Additionally, Ca was involved in other complexes that formed during sintering, which also promoted the discrepancy.

In addition, the lattice constant, crystal structure, and some other parameters (such as interplanar spacing) of β-Ti and Nb were similar, which made it difficult to distinguish them only by TEM. Therefore, it can be concluded that phases of spot 2 (Figure 8) also contained a mixture of β-Ti and Nb (probably only one). Some regions of titanium element in Figure 7b, which coincided with partially enriched regions of Nb (Figure 7d), indicated that Ti and Nb might form a solid solution. Wang et al. observed that composite powders of Ti-35Nb-2.5Sn/15HA were nearly spherical particles (about 300 nm), and HA was evenly distributed in the supersaturated β-phase Ti (Nb) solid solution [12]. Furthermore, it was found that the center of the enrichment zones was red and the surrounding zones were yellow or blue by comparing the enrichment regions of the elements. Therefore, this indicated that the corresponding concentration decreased gradually, and the conclusion of element diffusion was verified again during the sintering process. Balbinotti et al. reported that during the sintering process, Ti, O, P, and other elements were bonded by diffusion to form CaTiO_3_, Ti_x_P_y_, and other phases [14]. In addition, similar diffusion phenomena have been mentioned by Arifin et al. [33]. It can be seen that phases of point 2 (Figure 8) mainly contained CaO, CaCO_3_, CaZrO_3_, and so on, which confirmed the presence of the titanium–phosphorus compound previously analyzed. As described above, HA decomposes to produce CaO after sintering, and the free energy of Equation (12) was −90.811 kcal at 1000 °C, so the reaction could happen. CaZrO_3_ was also detected in the experiment by He et al. and was considered to be a reaction product of the chemical reaction (Equation (10)) [32].

(12) $$\mathrm{CaO}+C+O_{2}(g)={\mathrm{CaCO}}_{3}$$

Based on the mechanical properties and microstructure of the sintered nanocomposites, the strengthening and fracture mechanisms of the graphene-reinforced titanium matrix/nano-hydroxyapatite nanocomposites with different graphene content were analyzed. It was concluded that the strengthening mechanisms of graphene-reinforced titanium matrix/nano-hydroxyapatite nanocomposites with different graphene content were mainly second-phase strengthening, fine grain strengthening, dislocation strengthening, and dispersion strengthening. The diversity of elements added to the composites would improve the performance of the composites due to second-phase strengthening and dispersion strengthening [23]. Nano-level HA, lanthanum, and the preparation process could reduce the particle size of the composite. Furthermore, the fine grain boundaries could inhibit dislocation movement and enhance the mechanical properties of the composite. Some researchers found that during sintering cooling, residual stress at the interface was sufficient to produce dislocations due to the difference in thermal expansion coefficient between the graphene and titanium matrix [23,24]. The high dislocation density could release the residual stress of the matrix to achieve an enhancement effect [23,24]. Furthermore, graphene could also prevent dislocation movement and form a dislocation ring. Then, the effect of dislocation strengthening, dispersion strengthening, and load transfer [22,23,24] occurred in the sintered composite due to the Ovallon enhancement mechanism [24], which improved the mechanical properties of the composite with the increase of graphene content when the graphene content was lower. However, due to the relatively weak bonding between the layers, graphene was easy to slip when the graphene content was much higher. Then, the graphene would overlap and reunite due to the characteristics of graphene such as high specific surface area. This would further reduce its binding force and interface strength, resulting in the decrease of mechanical properties.

## 5. Conclusions

1. Graphene-reinforced titanium matrix/nHA composites were prepared by mechanical alloying and vacuum hot-pressing sintering. Results showed that during the high temperature sintering process, complex chemical reactions occurred, resulting in some new phases of nucleation such as Ca_3_(PO_4_)_2_, Ti_x_P_y_, and Ti_3_O. The new phases, which easily dropped off under the action of external force due to weak interfacial bonding with the substrate, could hinder the densification of sintering and increase the brittleness of the nanocomposites.
2. The shear fractures of the sintered composites were brittle fractures, and there were a lot of detritus and pores on the fracture surface of the composite in which their unique pore structure can give them the characteristic of ultralow density and large surface area for human body with structural and functional integration. There were many tiny particles around the holes, and the reaction and decomposition products of HA were distributed in the alloy matrix. It was believed that the weak interfacial bonding was caused by the difference between reaction products of HA and other materials such as the thermal expansion coefficient.
3. The microhardness, shear strength, and compressive strength of the composites had a decreasing trend with graphene contents of 0.5–1.5 wt %. This was caused by the agglomeration of graphene. Furthermore, the strengthening mechanisms of composites were mainly second-phase strengthening, fine grain strengthening, dislocation strengthening, and dispersion strengthening.

## Figures and Tables

**Table 1:** The EDS results (at %) for regions 1 and 2 in Figure 3b.

Region	O	P	Ca	Ti	Zr	Nb	La	Ta
1	46.98	13.70	29.21	6.76	2.70	0.52	---	0.13
2	69.18	8.38	14.71	3.39	2.42	1.73	0.01	0.18

**Table 2:** Mechanical properties of the nanocomposites sintered at 1000 °C.

Sample	Microhardness/HV		Compressive Strength/MPa	Shear Strength/MPa
	Front	Side		
0.5-GNFs	105.10	106.71	178.08	37.45
1.0-GNFs	75.39	70.63	140.76	30.60
1.5-GNFs	64.32	65.57	145.04	28.56

**Table 3:** The EDS results (at %) for regions A–E in Figure 13.

Region	O	P	Ca	Ti	Zr	Nb	La	Ta
A	61.91	1.97	5.83	0.76	1.58	27.83	0.06	0.05
B	41.34	1.46	0.61	55.19	0.40	0.94	---	0.06
C	81.94	0.16	1.52	2.12	0.76	0.28	13.21	---
D	41.81	1.69	4.37	10.75	0.02	2.81	---	38.54
E	21.91	1.40	2.18	9.98	0	1.88	---	62.85

**Table 4:** Grain size of some phases in the 0.5-GNFs nanocomposite calculated by XRD Pattern Processing and Identification software.

Element	Treatment Condition	2θ	Crystal Face	Grain Size/Å
α-Ti	milled for 5 h	40.147/38.440/35.135	(101)/(002)/(100)	371/333/311
	sintered at 1000 °C	39.818/37.777/34.885		441/470/399
Ta/Nb	milled for 5 h	38.440/69.664/55.625	(110)/(211)/(200)	333/272/275
	sintered at 1000 °C	38.613/69.795/55.724		305/204/249
